# Parent-reported Early Atypical Development and Age of Diagnosis for Children with Co-occurring Autism and ADHD

**DOI:** 10.1007/s10803-022-05488-0

**Published:** 2022-03-07

**Authors:** Willow J. Sainsbury, Kelly Carrasco, Andrew J. O. Whitehouse, Hannah Waddington

**Affiliations:** 1 Faculty of Education, Victoria University of Wellington, Murphy Building Level 8/Kelburn Parade, Kelburn, Wellington, 6140 New Zealand; 2 Telethon Kids Institute, Perth, Australia

**Keywords:** Co-occurring conditions, Diagnosis, Autism, Attention deficit hyperactivity disorder, Age of diagnosis, Atypical development

## Abstract

Autism and attention-deficit/hyperactivity disorder (ADHD) often co-occur. This survey of 288 New Zealand parents of children diagnosed with autism (n = 111), ADHD (n = 93), or both conditions (n = 84), examined the relations between age of diagnosis and early atypical development, the age specialist consultation was needed and types of specialists seen. Co-occurring autism and ADHD was associated with an earlier ADHD diagnosis and a later autism diagnosis. Parents of children with both diagnoses reported less atypical development in language and social behaviours compared to parents of children of autism, and this co-occurring group also experienced longer wait times to diagnosis, and saw more types of specialists prior to a diagnosis, than those with autism.

Autism spectrum disorder (hereafter referred to as autism) and attention-deficit/hyperactivity disorder (ADHD) are both neurodevelopmental conditions. Autism is characterised by impairments in social communication and interaction and rigidity of behaviour (American Psychiatric Association, 2013). Autism can be diagnosed at 1.5 years of age, but a meta-analysis found autism is usually diagnosed around 5 years of age (van’t Hof et al., 2021). ADHD is defined by a triad of core symptoms that include inattention, impulsivity, and hyperactivity which impairs a child’s functioning across multiple environments (American Psychiatric Association, 2013). In the United States of America, ADHD is usually diagnosed around 6 years (Visser et al., 2014). It is estimated that anywhere between 14 and 78% of children with autism also have ADHD (Mannion & Leader, 2013), making this one of the most common co-occurring psychiatric conditions (Antshel et al., 2016). The wide ranging estimates of co-occurrence may be attributed to differences in study population samples and whether researchers were measuring symptoms of ADHD or official clinical diagnoses (Mannion & Leader, 2013).

The study of co-occurring disorders is in its nascence. Multiple new models or approaches have been suggested, to conceptualise co-occurrence beyond discrete disease categories (Forbes et al., 2016; Rosen et al., 2018). In addition to a theoretical position, the study of autism + ADHD was not given attention because the Diagnostic and Statistical Manual of Mental Disorders (DSM) excluded the dual diagnosis of these conditions until the 5th edition (Kentrou et al., 2019).

Children with autism + ADHD often benefit from early intervention, which can decrease behavioural challenges and barriers associated with the conditions, and increase wellbeing and quality of life (Franz & Dawson, 2019; Risley et al., 2020). Access to early intervention during a sensitive developmental period can be hindered by a delay in diagnosis. Indeed, the presence of co-occurring ADHD may lead to a later diagnosis of autism compared to autism in the absence of ADHD (Jónsdóttir et al., 2011; Soke et al., 2018). Miodovnik et al. (2015) found that children with co-occurring autism and ADHD who received their ADHD diagnosis first, waited an average of 3 years longer for the additional diagnosis of autism. This was consistent across childhood and independent of the severity of symptoms. Wei et al. (2021), in a study involving 9539 children with autism + ADHD, demonstrated that, regardless of the order of the diagnoses, (i.e. ADHD first or autism first), the diagnosis of autism was delayed by an average of 1.8 years compared to the age of diagnosis in an autism only population.

There is comparatively little research into the age of ADHD diagnosis for children with autism + ADHD compared to those with ADHD only. Research suggests children with both diagnoses receive their ADHD diagnosis between 1 and 1.8 years earlier than ADHD only children (Sainsbury et al., in press). This may be because autism is developmentally apparent earlier and thus co-occurring ADHD could be diagnosed sooner due to earlier health seeking (Jensen & Steinhausen, 2015).

A greater understanding of the delay in autism diagnosis and early diagnosis of ADHD with co-occurring autism + ADHD requires an understanding of the diagnostic pathway for families. Previous research has found that parent-reported concerns about their child’s development between 1 and 2 years of age were predictive of an early autism diagnosis (Guinchat et al., 2012; Ozonoff et al., 2014). In particular, observed atypical development in language and social communication predicted earlier diagnosis (Ozonoff et al., 2014). In ADHD research parent-reported symptoms at 3 years predicted the diagnosis of ADHD at the expected age of 6 + years (Oerbeck et al., 2020). However, little is known about the effect of parent-reported atypical development in the first year of life on age of diagnosis for children diagnosed with autism + ADHD compared to autism or ADHD alone.

After noticing atypical development, parents often present their concerns to a specialist (Zablotsky et al., 2017). Waddington et al. (in press) found that, for children with a diagnosis of autism, the age at which parents sought specialist consultation for their child with autism was reduced by 2 months for each additional parent-reported domain of atypical development in the first year of life. Stevens et al. (2016) conducted a study to compare the timing of parental concerns between autism and autism + ADHD groups. They concluded that both the first reported parental concern and the age at which a specialist assessment was sought were later for an autism + ADHD group of children than for those in an autism only group. There is also evidence that the nature of the concern that a parent presents to a specialist might aid in an earlier diagnosis of autism when the presenting concern fits with a symptom of the subsequent diagnosis, as the concern triggers that particular diagnostic pathway (Matheis et al., 2017; Rosenberg et al., 2011).

Once diagnosed, there is the question of whether parents feel that either or both diagnoses fit with the ongoing observed behaviour of their child. Researchers have argued for the co-occurring diagnosis to be further defined by the more dominant diagnosis, such as autism (+ ADHD) or ADHD (+ autism) (Van Der Meer et al., 2012). There is a gap in the literature pertaining to which diagnosis best fits with the ongoing behaviour that parents notice. One hypothesis is that parents of the children in the co-occurring group, might report that their children’s observable behaviour is more aligned with the diagnosis more frequently given first; ADHD. This would fit with the hypothesis that the ADHD truly masks autism symptoms or is the more dominant observable behaviour (Davidovitch et al., 2015). In the ADHD only group and the autism only group, it is expected parents view the diagnosis as a more consistent summary of their child's behavioural symptoms.

The literature indicates that the timing, frequency and type of parent-reported atypical development and the nature and timing of specialist consultation might play a role in the age of an autism diagnosis (Zablotsky et al., 2017). Little is understood, however, about how patterns in these areas might compare across groups of autism only, ADHD only and a combined group. In particular an ADHD only group is often not included in comparative studies. This is a particularly important area of study given the hypothesis that children with both conditions resemble more closely early developmental ADHD patterns, rather than autism, which might account for the delay in autism diagnosis (Davidovitch et al., 2015; Soke et al., 2018).

This study aimed to build a clearer picture of why there might be differences in the timing of both autism and ADHD diagnosis when the two conditions co-occur, compared to when they are present in isolation. Specifically, a survey was conducted of New Zealand parents of children with autism only, ADHD only, and autism + ADHD to examine the influence of various aspects of the diagnostic pathway on the timing of both autism and ADHD diagnosis across these three groups. These variables were: (a) the number and type of parent-reported areas of atypical development in the first year of life; (b) the age at which parents felt specialist consultation was needed; (c) the behaviour that led to specialist consultation and whether this aligned with the child's subsequent diagnosis; (d) the wait time from when parents felt specialist consultation was needed to when a diagnosis was given; (e) the types of specialist seen before a diagnosis; (f) any additional diagnoses; and (g) parental agreement that their child’s behaviour matched their diagnosis.

## Methods

### Ethical Clearance and Informed Consent

Ethical approval for this study was provided by the Human Ethics Committee at Victoria University of Wellington, New Zealand [Approval number 28993]. Participation in the study was both voluntary and anonymous. Participants were asked not to include any information which would make them or their child identifiable. Participants were also informed that they did not have to answer any question and were given "prefer not to say" options.

### Participants

Parents of children with autism, ADHD, or autism + ADHD were invited to participate in this survey. Participants were eligible for inclusion if they were: (a) parents, legal guardians, or caregivers (hereafter, parents), (b) the child had a clinical diagnosis of autism, ADHD, or autism + ADHD, and specified the age of diagnosis (as reported by parents), (c) the child was aged 18 years or younger, and (d) the parents and child were living in New Zealand. Convenience sampling was used, as participants were recruited by an email sent by Autism New Zealand and ADHD New Zealand, as well as information about the study shared on various autism and ADHD social media groups. Participants were sent a link to the questionnaire within the advertisement.

### Materials

The survey was hosted on Qualtrics from the 15th of March until the 1st of June, 2021. Many of the questions used in this survey were based on those in The Australian Autism Biobank's Family History Questionnaire (Alvares et al., 2018). The survey was revised after consultation with a researcher and advocacy advisor at Autism New Zealand. The questionnaire had 25 questions separated into 5 sections related to: (a) demographic characteristics, (b) retrospective parent-reported atypical development, (c) first concerns, (d) specialists seen and additional diagnoses, and (e) age of diagnosis and fit of diagnosis from the parents' perspective.

The demographic characteristics section included questions about parent ethnicity, relationship to the child, highest education level, and total household income. Child demographic characteristics included gender, age, and the presence and birth position of siblings.

The atypical development section involved two questions. First, *"do you recall anything unusual about your child's development or behaviour during the first 6 months of his/her life?",* (referred to herein as parent-reported atypical development at ≤ 6 months) and "*Now think of the period between 7 and 12 months, can you recall anything unusual about your child's behaviour during that time?"* (referred to herein as parent-reported atypical development at 7–12 months). The parents then selected which of 30 areas of atypical development were retrospectively relevant to their child at each age. These were based on the areas identified by Waddington et al. (in press) and Guinchat et al. (2012). Some language was changed to be user-friendly, and categories that had no responses in Waddington et al. (in press) were not included. Areas were identified by parents of autistic children as very early signs of atypical development, but were not necessarily specific to the autism criteria (Guinchat et al., 2012). The social development and stereotyped/restricted behaviour domains are associated with the diagnostic criteria for autism, and the hyperactivity and lack of attention subdomains within the temperament domain are associated with the diagnostic criteria for ADHD. The 30 areas were subdomains of seven domains of atypical development: language, social, stereotyped behaviour, motor, behaviour/temperament, medical, and abnormal physiological function. Parents could also select “other” and provide a description of any atypical development not covered in the list. These “other” atypical development descriptions were then coded in their entirety by WS (a researcher and an educational psychologist) and 20% were also independently coded by a research assistant. Domain and subdomain level agreement was calculated using the formula agreements/(agreements + disagreements) × 100, Domain-level agreement was 92.9% at ≤ 6 months and 96.2% at 7–12 months, sub-domain level agreement was 97.2% and 98.0% respectively.

The next section related to type and timing of the parental concern(s) that led them to seek specialist help. Parents were asked how old the child was when they first had concerns that warranted the need to see a specialist and to describe what particular concerns led to seeking specialist help in an open-text box. The concerns were then coded by WS (a researcher and an educational psychologist) according to whether the concerns fit with a DSM 5 description of the particular diagnosis(es) the child obtained. The coding was based on ADHD concerns, autism concerns or behaviour/other category. A further 30% were coded by KC (a researcher and an educational psychologist) with an 88% interrater agreement.

The next section related to specialists seen, additional diagnoses and age of diagnosis. Parents were asked to select the types of specialists/professionals seen before receiving a diagnosis from a list and an "Other" option with text provided. The next question asked whether their child was diagnosed with autism, ADHD, or autism + ADHD. Depending on parents’ responses, they were directed to the child’s age of diagnosis for either autism only, ADHD only, or an age for both diagnoses. The last three questions of this section asked, "What diagnosis best described the behaviours of your child?" with the diagnostic options of autism and ADHD with main symptoms in brackets, a "both" and a "neither" option. A further two questions about any additional diagnoses with options including sensory processing disorder, epilepsy/seizures, oppositional defiant disorder, intellectual disability, global delay, learning disability and "other" and finally whether a misdiagnosis had occurred. A final section asked four open-ended qualitative questions, which are not included in the analyses.

### Data Analysis and Management

Data analysis was performed using IBM SPSS Statistics software (version 26). A chi-squared test of independence with Bonferroni correction determined whether there were significant differences across diagnostic groups for gender, ethnicity, household income, highest education and absence or presence of siblings. A Kruskall Wallis test was used to analyse the age of participants across diagnostic groups.

Household income data was collapsed from 13 $NZ 10,000 increments to four $NZ 50,000-increments. In the highest level of education question many of the "Other" responses related to diplomas or trade qualifications, thus, a new category was added to encompass these responses. It was not possible to do an analysis for parent relationship to child due to having too few participants in any other category except mothers. Categories with fewer than 10 participants in each group were considered for exclusion or merging into a wider category to increase the reliability of group measures in statistical analysis. Excluded from statistical analysis of gender were 'non-binary' (n = 6) and 'prefer not to say' (n = 2) and excluded from analysis of siblings was the category of "twins" (n = 9). None of the "Other" ethnicities reached a threshold for analysis and were thus grouped together. Each participant’s ethnicity was categorised as ‘Māori’ (the indigenous people of New Zealand) if one of their stated ethnicities was Māori.

Non-parametric statistical tests were also used to answer the research questions as the data was not normally distributed. Mann Whitney-U tests were used to compare the age of autism and ADHD diagnosis, time to diagnosis, and the alignment of parents’ description of behaviour with symptoms of the child's subsequent diagnosis. The autism + ADHD group was not included in the latter analysis due to the presenting behaviour description requiring symptom agreement of both an autism and ADHD diagnosis and most parents only described one presenting behaviour. Kruskall-Wallis tests were used to compare differences across the three diagnostic groups in terms of: (a) age of children at the time of the survey; (b) total number of parent-reported atypical development; (c) age of from when specialist was sought; (d) age when specialist was sought; (e) the number of different types of specialists seen, (f) the total number of additional diagnoses across the three groups. The Kruskal–Wallis test presented the post-hoc p values in relation to each group combination and are reported. Chi-squared tests were used to compare differences across the three diagnostic groups in (a) atypical development in each domain at ≤ 6 and 7–12 months; (b) alignment between parents’ perceptions of the child's behaviour presented to a specialist and the child's subsequent diagnosis; (c) alignment between diagnosis and parent perceptions of child's ongoing behaviour; and (d) additional diagnoses. To control for multiple comparisons, a Bonferonni correction was used for post-hoc tests by multiplying all *p* values by 3.

## Results

### Sample Characteristics

The parents of 355 children participated in the survey. Of these, 52 parents did not report their child's diagnosis, four parents reported that their children were over 18, and 11 only completed the demographic questions, and were thus excluded. This resulted in a final sample of 288 parents of children diagnosed with either (1) autism; (2) ADHD; or (3) autism + ADHD. Table 1 provides the demographic characteristics for these participants both overall and across diagnostic groups. In terms of the overall sample, the children were predominantly male with a mean age of 113 months (9.5 years). Mothers were more likely to have completed the survey. The parents who completed the survey were more commonly NZ European, had completed university and had a household income between $NZ 50,000–99,000.

**Table 1:** Child and parent demographic characteristics (n = 288)

Demographic characteristic	Total n (%)/mean (months) (SD)	Autism only n (%)/mean (months) (SD)	ADHD only n (%)/mean (months) (SD)	Autism + ADHD n (%)/mean (months) (SD)	Missing data
Participant numbers Child gender	288	111	94	83	
Male	215 (74.7)	81 (73.0)	67 (71.3)	67 (80.7)	
Female	65 (22.6)	26 (23.4)	24 (25.5)	15 (18.1)	
Non-binary	6 (2.1)	3 (2.7)	2 (2.1)	1 (1.2)	
Prefer not to say	2 (0.7)	1 (0.3)	1 (0.3)	0 (0)	
Child age	113.4 (44)	97.9 (49)	118.3 (36)	128.7 (37)	
Age specialist needed		32.7 (27)	56.1 (33)	43.8 (26)	5
Child age when diagnosed with autism		63.1 (40)		87.6 (38)	0
Child age when diagnosised with ADHD			90.0 (28)	81.5 (26)	2
Parent relationship to child					
Biological mother	273 (94.8)	107 (96.4)	89 (94.7)	77 (92.8)	
Biological father	7 (2.4)	2 (1.8)	4 (4.3)	1 (1.4)	
Legal guardian/caregiver	8 (2.8)	2 (1.8)	1 (1.1)	5 (6.0)	
Siblings of child					
No siblings	31	9	13	9	
Younger siblings	149	58	55	36	
Older siblings	170	69	45	56	
Twins	9	4	2	3	
Parent ethnicity					1
NZ European (Pākehā)	200 (69.4)	70 (63.1)	70 (74.5)	60 (72.3)	
Māori	64 (22.2)	29 (26.1)	14 (14.9)	21 (25.3)	
Other: (Pacific People, Asian, European, South African, American, South American, Middle Eastern, African)	23 (8.0)	12 (10.8)	9 (9.6)	2 (2.4)	
Parent highest education					
< 12 years	36 (12.5)	14 (12.6)	14 (14.9)	8 (9.6)	
12 years	75 (26.0)	34 (30.6)	20 (21.3)	21 (25.3)	
Diploma/Trade	23 (8.0)	7 (6.3)	10 (10.6)	6 (7.2)	
University Prefer not to say/other (not specified)	131 (42.4) 23 (8.0)	48 (43.2) 8 (7.2)	42 (44.6) 8 (8.5)	41 (49.4) 7 (8.4)	
Household income $NZ					
< $50,000	59 (20.5)	20 (18.0)	11 (11.7)	28 (33.7)	
$50,000–$99,999	105 (36.5)	44 (39.6)	39 (41.5)	22 (26.5)	
$100,00–149,999 $150,000 or more	52 (18.1) 50 (17.4)	19 (17.1) 18 (16.2)	17 (19.1) 19 (20.2)	16 (19.3) 13 (15.7)	
Prefer not to say	22 (7.6)	10 (9.9)	8 (8.6)	4 (4.8)	

Parent education and parent ethnicity relate to the parent who completed the survey. Pākehā is an indigenous term used for New Zealanders of European descent

Chi-squared tests indicated that there were no significant differences across diagnostic groups in terms of highest level of education [X^2^ (6, n = 265) = 4.40, *p* = 0.623], ethnicity [X^2^ (4, n = 288) = 9.11 *p* = 0.058] or gender [X^2^ (2, n = 280) = 1.69, *p* = 0.429]. A significant difference was found for the association between the diagnostic group and having a younger sibling [X^2^ (2, n = 288) = 7.73, *p* = 0.021] with post-hoc testing indicating a lower proportion of younger siblings in the ADHD group compared to the autism or co-occurring group. There were no other significant differences in the likelihood of having no siblings [X^2^ (2, n = 288) = 1.74, *p* = 0.420] or older siblings [X^2^ (2, n = 288) = 4.06, *p* = 0.131] across diagnostic groups. There were significant differences in family income across diagnostic groups [X^2^ (6, n = 266) = 14.66, *p* = 0.023]. Post-hoc testing indicated that the autism + ADHD group had a smaller proportion of participants in the $NZ50,000–99,999 group compared to both the autism and ADHD only groups.

A Kruskal–Wallis test determined that there were significant differences in child age across diagnostic groups [*H*(2) = 27.331, *p* < 0.001]. Post-hoc testing determined that the age of the autism group was significantly lower compared to those with both autism + ADHD and those diagnosed with ADHD, *p* < 0.001 and *p* = 0.045, respectively. The age of ADHD children was also significantly younger than the autism + ADHD group (p = 0.040).

### Age of Diagnosis

Mann–Whitney U tests indicated that an ADHD diagnosis was given significantly earlier for the autism + ADHD group (Mdn = 79.0) (months) than for the ADHD only group (Mdn = 96.2), U (n = 83, n = 92) = 3067.00, z = − 2.25, *p* = 0.025, while an autism diagnosis was given significantly earlier for the autism only group (M = 81.0) than for the autism + ADHD group (Mdn = 119.6), U (n = 111, n = 83) = 2771.50, z = − 4.75, *p* < 0.001.

### Atypical Development at ≤ 6 Months

The percentage of participants reporting atypical development in each domain and subdomain across diagnostic groups is reported in Table 2.

**Table 2:** Percentage of parents reporting atypical development in each domain and subdomain across diagnostic groups when their children were ≤ 6 and 7–12 months (n = 288)

Domain-/Subdomain	≤ 6 months			7–12 months		
	Autism only	ADHD only	Autism + ADHD	Autism only	ADHD only	Autism + ADHD
Reported at least one area of atypical development across domains	59.5	54.3	60.2	82.0**	73.4**	86.9**
Language	**23.4**	**12.8**	**24.1**	53.2**	19.1**	43.4**
Delayed speech/vocalizations	17.1	9.6	16.9	47.7	18.1	37.3
No speech/vocalizations	11.7	4.3	9.6	13.5	4.3	9.6
Poor language comprehension	15.3	7.4	10.8	33.3	8.5	21.7
Loss of language	2.7	0.0	3.6	15.3	2.1	10.8
Social development	**30.6**	**17.0**	**22.9**	59.5**	23.4**	45.8**
Delayed social communication	15.3	8.5	12.0	37.8	10.6	26.5
No social communication	8.1	2.1	6.0	21.6	1.1	10.8
Gaze abnormalities	18.0	7.4	13.2	34.2	10.6	31.3
Poor social interaction	15.3	10.6	14.5	38.7	17.0	31.3
Lack of response to social stim	21.6	5.3	10.8	41.4	8.5	26.5
Stereotyped/restricted behavior	**38.7**	**30.9**	**34.9**	64.0*	47.9*	69.9*
Stereotyped movements	20.7	5.3	12.0	41.4	12.8	24.1
Need for routine/rituals	21.6	14.9	20.5	43.2	26.6	38.6
Stereotyped/restricted interests	9.9	3.2	10.8	40.5	11.7	32.5
Preoccupation with object	14.4	7.4	10.8	40.5	19.1	28.9
Hypersensitivity	25.2	14.9	21.2	48.6	23.4	51.8
Hyposensitivity	9.0	2.1	4.8	18.0	5.3	13.3
Motor development	**31.5**	**20.2**	**26.5**	45.9*	25.5*	41.0*
Motor delay	20.7	8.5	16.9	37.8	20.2	31.3
Hypotonia	6.3	3.2	10.8	9.0	4.3	15.7
Hypertonia	4.5	2.1	2.4	8.1	2.1	3.6
Swallowing/Sucking	14.4	13.8	19.3	11.7	7.4	13.3
Behavior/Temperament	**37.8**	**39.4**	**44.6**	**56.8**	**57.4**	**71.1**
Lack of attention	7.2	10.6	14.5	21.6	18.1	34.9
Hyperactivity	6.3	20.2	16.9	33	20.7	43.4
Passivity	9.0	4.3	9.6	9.0	4.3	10.8
Tantrums/opposition	11.7	7.4	12.0	36.9	22.3	39.8
Difficult to soothe	26.1	24.5	33.7	32.4	27.7	39.8
Aggression/violence	3.6	2.1	3.6	10.8	2.1	14.5
Self-harm	3.6	0.0	1.2	10.8	1.1	4.8
Extreme attachment to caregiver	20.7	10.6	19.3	30.6	20.2	37.3
Medical issues	**12.6**	**19.1**	**10.8**	**15.3**	**19.1**	**15.7**
Another disorder	1.8	4.3	1.2	0.9	5.3	1.2
Sickness	11.7	17.0	9.6	14.4	14.9	14.5
Abnormal physiological function	**34.2**	**36.2**	**42.2**	**56.8**	**48.9**	**50.6**
Sleeping	27.0	35.1	39.8	41.4	45.7	49.4
Feeding	18.0	11.7	18.1	35.1	13.8	20.5

Items in bold are domains while indented, non-bolded items are subdomains and asterisks indicate p value (*p = 0.05 **p = 0.001)

A Kruskal–Wallis test was used to compare total number of domains of atypical development indicated by parents and there was no statistically significant difference between groups in atypical development [*H*(2) = 1.119, *p* = 0.572]. Chi-square test for the presence or absence of any area of atypical development did not significantly differ between the three groups, [X^2^ (2, n = 288) = 809, *p* = 0.667].

Chi-square tests revealed no significant difference across diagnostic groups in domains of language [X^2^ (2, n = 288) = 4.73, *p* = 0.94]; social [X^2^ (2, n = 288) = 5.25, *p* = 0.73]; stereotyped behaviour [X^2^ (2, n = 288) = 1.39, *p* = 0.50]; motor [X^2^ (2, n = 288) = 3.36, *p* = 0.19], behaviour [X^2^ (2, n = 288) = 0.95, *p* = 0.62]; medical issues X^2^ (2, n = 288) = 2,88, *p* = 0.24); or abnormal function [X^2^ (2, n = 288) = 1.34, *p* = 0.51].

### Atypical Development at 7–12 Months

A Kruskal–Wallis test was used to compare total number of domains of atypical development indicated by parents and there were significant differences between groups [*H*(2) = 16.149, *p* < 0.001]. Bonferroni-adjusted pairwise comparisons determined that ADHD had significantly fewer parent-reported areas of atypical development than autism (*p* < 0.001) and autism + ADHD (*p* = 0.014) with no significant difference between autism and autism + ADHD (*p* = 1.000). Chi-square test for the presence or absence of any area of atypical development did not significantly differ between the three groups, (X^2^ (2, n = 288) = 5.25, *p* = 0.073).

There were no significant differences in domains of behaviour [X^2^ (2, n = 288) = 4.89, *p* = 0.087], medical issues [X^2^ (2, n = 288) = 0.62, *p* = 0.732] and abnormal function [X^2^ (2, n = 288) = 2.50 *p* = 0.287]. There were significant differences in the language domain [X^2^ (2, n = 288) = 225.52, *p* < 0.001]. There were a higher proportion of parents reporting atypical development in the language domain if their child had autism than autism + ADHD, and a significantly lower proportion for the ADHD group compared to both autism and autism + ADHD groups*.* There were also significant differences across diagnostic groups in the social domain [X^2^ (2, n = 288) = 27.08, *p* < 0.001], with the a higher proportion in the autism group reporting atypical social development than the ADHD and autism + ADHD groups and a lower proportion in the ADHD group than all other groups.

There were significant differences in the stereotyped behaviour domain [X^2^ (2, n = 288) = 9.88, *p* = 0.007], with a greater proportion of parents in the autism + ADHD group reporting atypical stereotyped behaviour than the ADHD group, and the ADHD group had a lower proportion than the autism and the autism + ADHD groups. There were also significant differences in the motor domain [X^2^ (2, n = 288) = 9.95, *p* = 0.009] with a higher proportion of parents reporting atypical motor development in the autism group and a lower proportion of parents reporting atypical development in the ADHD group.

### Age Specialist Needed

A Kruskal–Wallis H test indicated that the age (months) at which parents felt specialist help was needed differed between the three diagnostic groups (*H*(2) = 36.647, *p* < 0.001). Post-hoc analysis determined that the age a specialist was needed was significantly lower in those with autism compared to those with both autism + ADHD and those diagnosed with ADHD alone, [*H*(2) = 17.040, *p* < 0.001] and [*H*(2) = 35.759, *p* < 0.001] respectively. The age a specialist was needed did not differ between the autism + ADHD and ADHD groups [*H*(2) = 2.290, *p* = 0.391].

Further analysis of the specific behaviours which parents report led them to seek specialist help are shown in Table 3. The behaviours parents presented were coded for whether they aligned with the child's subsequent diagnosis. The differences in the agreement of type of presenting behaviour of the subsequent diagnosis was significant X^2^ (2, n = 288) = 96.45, *p* < 0.001. Parents of children with autism most frequently presented with autistic behaviour(s). The ADHD and the autism + ADHD group showed far greater inconsistency with a greater reporting of behaviours that do not fit with an autism or an ADHD diagnosis.

**Table 3:** Alignment between behaviours that led parents to seek specialist help and diagnosis

Diagnostic groups	Behaviours related to Autism only (n/%)	Behaviours related to ADHD only (n/%)	Behaviours related to Autism + ADHD (n/%)	"Other" behaviours
Autism	91 82%	0 0%	0 0%	20 18%
ADHD	16 17%	39 41.5%	1 1.1%	38 40.4%
Autism + ADHD	32 38.6%	5 6%	11 13.3%	35 42.2%

### Wait Time from Age Specialist Needed to Age of Diagnosis

Mann–Whitney U tests were used to examine the time (months) between when a specialist was needed to diagnosis for ADHD and autism groups compared to the autism + ADHD group. Analysis indicated that the wait time from age parents felt a specialist was needed to diagnosis for ADHD only group and the co-occurring group was not significant U (n = 162) = 3 506.00, z = 0.763 *p* = 0.445. The wait time from specialist contact to diagnosis for the autism only and autism + ADHD group were significant with the autism only group (M = 82.83) waiting significantly less time for an autism diagnosis than an autism + ADHD group (M = 109.53), U(n = 188) = 5582.50, z = 3.345, *p* = 0.001.

### Alignment of Parents’ Description of Behaviour with the Child's Subsequent Diagnosis and the Effect on Time to Diagnosis

A further Mann–Whitney U analysis examined whether the type of behaviour described by parents as motivating specialist help aligned with a symptom of the child's subsequent diagnosis and whether this affected time to diagnosis. In the autism only group, parents who described a symptom of autism as motivating them to seek specialist help waited significantly less time for a diagnosis than parents of autism only children who presented with concerns that did not indicate a symptom of autism, U (n = 91, n = 20) = 616.50, z = − 2.254, *p* = 0.024. The same was true within the ADHD only group. Parents who presented with concerns that indicated a symptom of ADHD waited significantly less time than parents who presented with concerns that did not indicate a symptom of ADHD, U (n = 37, n = 55) = 444.00, z = − 4.572 *p* < 0.001.

### Number of Types of Specialists Seen

A Kruskal–Wallis test was used to compare the number of different types of specialists seen between the three diagnostic groups, determining that at least one group differed [*H*(2) = 14.776, *p* = 0.001]. Bonferroni-adjusted pairwise comparisons determined that the number of types of specialists seen was significantly higher in those with autism + ADHD compared to those diagnosed with ADHD alone and autism alone (*p* < 0.001 and *p* = 0.044, respectively). There was no difference in the number of types of specialists seen between the ADHD and autism only groups (*p* = 0.340).

### Total Additional Diagnoses

A Kruskal–Wallis test was used to compare the number of additional diagnoses between the three diagnostic groups, determining that no group significantly differed; H(2) = 4.86, p = 0.089. However, certain diagnoses had a significant correlation with particular diagnostic groups. Oppositional defiance disorder was more likely to be diagnosed in addition to ADHD than autism and autism + ADHD [X^2^ (2, n = 288) = 26.55 *p* =  < 0.001] and sensory processing disorder was more likely to be diagnosed with autism than ADHD and autism + ADHD [X^2^ (2, n = 288) = 13.21 *p* < 0.001]. Intellectual disability (ID) was not significant across the groups (n = 9 ADHD only; n = 15 Autism; n = 15 Autism + ADHD); [X^2^ (2, n = 288) = 2.72 *p* = 0.257].

### Diagnosis and Parent Perceptions of Child's Behaviour

There were no significant differences between groups about whether parents believed that the diagnosis explained their child's behaviour [X^2^ (2, n = 288) = 1.75 *p* = 0.417]. Within the ADHD group, 25% of parents disagreed that ADHD was the best diagnostic description for their child's behaviour and selected both autism + ADHD as a better explanation. Within the autism group, 20.7% disagreed that autism was the best diagnostic description for their child's behaviour and selected both except for 3 parents, who opted for neither. Within the co-occurring group, 16.9% disagreed that autism + ADHD was the best diagnostic description for their child's behaviour and selected either ADHD (n = 8) or autism (n = 7).

## Discussion

This study sought to understand similarities and differences in early childhood diagnostic experiences for children with diagnoses of autism, ADHD or both. In line with previous research, ADHD diagnoses were significantly earlier for the autism + ADHD group compared to the ADHD only group, and autism diagnoses were significantly later for the autism + ADHD group compared to the autism only group (Sainsbury et al., in press). The results provide further data that children with autism + ADHD may present with a different and more complex diagnostic picture. This presentation of retrospective parent-reported atypical development appeared as a "half-way" point with higher reported atypical development than an ADHD group, but lower than an autism group. Along the diagnostic pathway the co-occurring group resembles more closely the characteristics of an ADHD group, such as with timing of when a specialist was needed. In other measures, an autism + ADHD group presented with neither autism nor ADHD patterns such as parents' descriptions of the concern that led to specialist help being more ambiguous, possibly contributing to the co-occurring group having longer wait times and seeing more types of specialists between seeking help to diagnosis than the autism only or ADHD only groups.

The parent reported observation of atypical development demonstrated that under 6 months there were no significant differences across diagnostic groups, but by 12 months statistically significant differences were occurring. The significant differences between increased atypical development reported in the autism only group compared to the ADHD group, was to be expected. Core autism behavioural features of language, stereotyped behaviour and social atypical development were significantly more reported in the autism group than the ADHD group which fits with diagnostic models that suggest ADHD symptoms are more apparent in later childhood and autism symptoms are observable at 12 months (Ozonoff et al., 2014). In Wallisch et al. (2020)'s study of a parent population who had 3–5 year olds diagnosed with multiple diagnoses, they found similar patterns of atypical parent reported concerns with language, and social atypical development reported less in an ADHD group compared to an autism group. Early signs of ADHD are usually considered to occur during toddler years, however, recent research is finding that, like autism, atypical development is seen in the first year of life with feeding and sleeping difficulties and motor difficulties, in children subsequently diagnosed with ADHD (Athanasiadou et al., 2020). Parents of children with ADHD did report sleeping difficulties at similar rates to the other diagnostic groups.

The autism + ADHD diagnostic group had fewer areas of reported atypical development than the autism group but more than the ADHD group. The prevalence of atypical language and social development in the first year of life appeared to be markedly less for autism + ADHD group. Atypical language development is a known trigger for early health seeking and less atypical development in this area is likely to contribute to a delay in an autism diagnosis (Zablotsky et al., 2017). However, this trend was reversed with stereotyped behaviour, whereby parents of autism + ADHD children reported higher atypical development in this area than autism and ADHD children. This might be a possible focus for diagnostic identification in children with co-occurring autism and ADHD.

The average age when parents first sought a specialist differed significantly. The delay in diagnosis may start, in part, with a delay in parents of children with autism + ADHD seeking specialist help compared to an autism only group (Stevens et al., 2016). The age at which parents of the co-occurring group sought specialist help was significantly later than the autism only group, but not statistically different from the ADHD group. In this way the co-occurring group fits an ADHD pattern. The delay in help seeking for these groups might be due, in part, to the nature of the child's behaviour in that it might be attributed to temperament, behavioural difficulties or parenting rather than requiring specialist intervention (Wallisch et al., 2020; Yamauchi et al., 2015).

Parents presenting with a specific symptom-related behaviour which corresponds directly to a particular diagnosis are likely to trigger a particular diagnostic pathway (Becerra-Culqui et al., 2018). The autism group was the most reconcilable in terms of presenting behaviours that described a symptom of autism. Analysis demonstrated that when parents described behaviour that was consistent with a symptom of a particular diagnosis in either the ADHD or the autism group, those families had a shorter wait time to diagnosis than parents who presented with a less diagnostic-specific behaviour.

Parents of the co-occurring group described presenting behaviours to specialists which did not fit with key symptoms that might trigger a diagnosis of either disorder, but rather the most common concerns parents described appear to be "other", including behaviour, then autism and then ADHD symptoms. This might result in a less clear referral to a specialist or a referral to a specialist who does not have a specialty in autism or ADHD diagnosis. Evidence of this was seen in the increased number of types of specialists that the co-occurring group saw before receiving a diagnosis. Eggleston et al. (2019)’s study of parents’ experiences of their child receiving an autism diagnosis found that co-occurring ADHD was also predictive of a higher number of professionals consulted prior to diagnosis, (Eggleston et al., 2019). It is also interesting that there were a lower number of parent-described ADHD behavioural symptoms that initiated specialist help in the co-occurring group, given that an ADHD diagnosis is often made earlier compared to an ADHD only group, and ADHD is more commonly diagnosed first in a co-occurring group (Kentrou et al., 2019). Thus, it was hypothesized that parents would describe behaviour that was more likely to flag ADHD symptoms. However, in some ways this group did resemble the ADHD group because of the high number of "other" behaviours. Primarily behavioural concerns have been shown to be associated with delays in seeking specialist help, possibly due to parents thinking the behaviours are due to the temperament of the child or perceived as a parenting issue (Ghanizadeh, 2007; Yamauchi et al., 2015).

This study elucidated further where along the diagnostic pathway delays in diagnosis might be occurring. Stakeholders in the diagnosis and treatment of children might consider that parents of children with autism + ADHD often present with later behavioural and other types of concerns, and with less parent-reported atypical development of language and communication than an autism only group. A further consideration might also be that parents presenting with concerns around behaviour and unclear referrals may warrant a more direct referral to a multidisciplinary team rather than referrals to a number of types of individual practitioners. Referrals that trigger specific developmental symptoms of a particular diagnosis are likely to encounter a more straightforward diagnosis pathway. In addition, specialists might consider that a first diagnosis of ADHD should not preclude further assessment of autism, or that autism should at least not be ruled out. Specialist might also want to consider discussing with parents, whose children are first diagnosed with ADHD, the key symptoms of autism so that parents feel empowered to return for the possibility of an autism diagnosis sooner. These suggestions have the potential to reduce the delay in diagnosis for autism + ADHD children and improve the diagnosis and treatment for children and their families.

One limitation of this study is that it did not use an epidemiological sample, and may not be representative of the broader population of these groups. A more educated and homogenous sample of society often answer online surveys (Eggleston et al., 2019). However, the mean data of the survey represented a cross section of New Zealand based on highest education attainment and the household income of the sample (OECD, 2019). In terms of ethnicity, Māori, the indigenous population of New Zealand, are adequately represented in this sample (Tupou et al., 2021).

The categories of diagnosed children representing autism, ADHD and autism + ADHD are not static, and it is possible that children in the single diagnosis categories will, at some point in the future, move into the co-occurring group. However, this possible movement does not detract from the snapshot in time of these diagnoses and how they might relate to atypical development, timing and type of parental concerns, diagnosis experience and current diagnosis of the child. To enhance the level of evidence further studies might also look at the risks of mis-diagnosing co-occurring ASD + ADHD. A greater understanding of any undesirable consequences in giving a younger child a co-occurring diagnosis should be addressed.

There are limitations for parents self-reporting. The first concerns that led to specialist help also has a recall bias, particularly once diagnosis is known, and parents might retrospectively recall concerns that fit with the diagnosis. The survey uses parental report as the basis for diagnostic criteria, which had not been clinically confirmed. Miodovnik et al., (2015), however, cite research in both autism and ADHD areas that found convergent validity between parent reported ADHD diagnosis data from insurance claims and autism diagnosis data form nationally representative surveys. In addition, data about first concerns and diagnostic history were collected at one point in time for children whose ages ranged from 2 to 18 years of age. All parents were asked to reflect over time, and parents of the oldest children needed to reflect over the longest period which might create the possibility of differential recall biases as it relates to the parents’ report, particularly for the co-occurring diagnostic category with, on average, older children than the autism or ADHD group. A further consideration is differentiating between early atypical concerns only 6 months apart which is retrospectively remembered, and therefore, the data might not accurately reflect the progression of atypical development in this early year of life.

## Conclusions

The study found that the diagnosis of autism in an autism + ADHD population is delayed. A delayed diagnosis is significant for this high need population as it reduces the opportunities for early intervention. The delay in diagnosis may be due to parents noticing less atypical development in language and social behaviours, the presentation of less autism-specific concerns to a specialist and at a significantly later time than an autism only group. A lack of flagging for a diagnosis potentially might result in multiple referrals to different specialists and possibly a longer diagnostic pathway. These factors may contribute to a delay in an autism diagnosis for an autism + ADHD child.
