# Correction to: Parent-reported Early Atypical Development and Age of Diagnosis for Children with Co-occurring Autism and ADHD

**DOI:** 10.1007/s10803-022-05555-6

**Published:** 2022-04-06

**Authors:** Willow J. Sainsbury, Kelly Carrasco, Andrew J. O. Whitehouse, Hannah Waddington

**Affiliations:** 1 Faculty of Education, Victoria University of Wellington, Murphy Building Level 8/Kelburn Parade, Kelburn, Wellington, 6140 New Zealand; 2 Telethon Kids Institute, Perth, Australia

## Correction to: Journal of Autism and Developmental Disorders 10.1007/s10803-022-05488-0

In this article the original date was 2018 has been listed as 2021 with an additional author in the reference.

This has been corrected with this erratum.

The correct reference is given below.

Wei, H.-T., Hsu, J.-W., Huang, K.-L., Bai, Y.-M., Su, T.-P., Li, C.-T., Lin, W.-C., Tsai, S.-J., Pan, T.-L., & Chen, T.-J. (2018). Timing of the diagnoses of attention deficit hyperactivity disorder and autism Spectrum disorder in Taiwan. Journal of Autism and Developmental Disorders, 1–8.

